# Identification of differentially-expressed of Olfactomedin-related proteins 4 and COL11A1 in Iranian patients with intestinal gastric cancer 

**Published:** 2017

**Authors:** Asma Dabiri, Kaveh Baghaei, Mehrdad Hashemi, Shekoofeh Sadravi, Habib Malekpour, Manijeh Habibi, Farhad Lahmi

**Affiliations:** 1 *Department of Genetic, Tehran Medical Science Branch, Islamic Azad University, Tehran, Iran*; 2 *Basic and Molecular Epidemiology of Gastrointestinal Disorders Research Center, Research Institute for Gastroenterology and Liver Diseases, Shahid Beheshti University of Medical Sciences, Tehran, Iran.*; 3 *Department of Genetic, Tehran Medical Branch, Islamic Azad University, Tehran, Iran.*; 4 *Department of Agronomy and Natural Resources, University of Tehran, Tehran, Iran*; 5 *Gastroenterology and Liver Diseases Research Center, Research Institute for Gastroenterology and Liver Diseases, Shahid Beheshti University of Medical Sciences, Tehran, Iran*; 6 *Behbood Gastroenterology and Liver Diseases Research Center, Shahid Beheshti University of Medical Sciences, Tehran, Iran*

**Keywords:** Olfactomedin, Gastric cancer, COL11A1

## Abstract

**Aim:**

Due to limited information on these genes and to a better understanding of common biomarkers associated with cancer of the digestive tract routes, we aim to evaluated expression level of Olfactomedin4 (OLFM4) and (pro)collagen11A1/COL11A1 genes in people with gastric cancer in Iran.

**Background:**

Gastric cancer is one of the main cause of cancer death. The early prognosis of gastric cancer is still a matter of debate. Human olfactomedin4 (OLFM4) is a glycoprotein that generally known as the antiapoptotic protein. (pro) collagen11A1/COL11A1 codes for the alpha-1 subunit of type XI collagen which exists in extracellular minor fibrillar collagen. In most cases, OLFM4 and COL11A1 are found to be up-regulated in many types of human cancers including gastric cancer.

**Methods:**

35 tissue samples were collected including 25 sample of patients with intestinal gastric cancer and 10 healthy controls. Expression level of OLFM4 and COL11A1 genes identified by using RGQ software. For analysis of real time-PCR products, Rotor-Gene Q series software was used.

**Results:**

Our finding showed that expression level of OLFM4 was significantly upregulated and COL11A1 did not show any significant difference in expression level in Iranian population with gastric cancer samples compared with those in normal samples.

**Conclusion:**

The results recommend that expression profiling of OLFM4 can be used for diagnosis of gastric cancer, and OLFM4 seems to be used as a biomarker for the diagnosis of gastric cancer. Regarding to our result, unlike some studies, COL11A1 did not show any significant difference between normal and tumor tissue which could explain ethological role in distribution of gastric cancer.

## Introduction

 Annually a large number of people lose their lives in avoidable and curable cancers(1). Gastric cancer (GC) is one of the main causes of cancer mortality among various cancers(2). According to investigations in 2012, GC is the fifth most frequent cancer and the third leading cause of cancer death across the world(3). GC also called stomach cancer is a disease in which cells of inner lining of the stomach are divided out of control and finally would be transformed into a tumor(4). GC rates are various in different places. The incidence of cancer varies by region, so foreign studies may not be extensible inside the country. According to the latest studies, GC is the seventh cause of all deaths in Iran. Also, it is the first cause of cancer death in Iranian men and the second cause of cancer death in Iranian women after breast cancer(3).

GC is a major health problem that environmental and genetic factors involved in its genesis (5). This cancer has poor symptoms in early stages. Thus the GC is almost diagnosed when it is in the final stages(6). Therefore, early detection and screening program is critical to improve prognosis in GC patients (7). 

Olfactomedin 4 (OLFM4), which also known as GW112, HGG1, pDp4, hOlfD is one of the members of olfactomedin protein family in mammalians(8). Researches have shown that carboxyl end of olfactomedin protein has about 250 amino acid in common with many other proteins, so-called olfactomedin domain (9). Mammalians have at least 13 proteins containing olfactomedin domain, which is important for neural crest formation, ventral patterning, cells connection, cell cycle regulation and tumorigenesis (10).

OLFM4 expression is regulated by NF-kappa B and AP-1transcription factors (11). It was determined that these secretory proteins have interaction with surface proteins such as lectins, Concanavalin-A(12). Recent studies show OLFM4 role in appearance and development of various malignances(13) such as Gastrointestinal cancer(14). Induction of OLFM4 in cancer cells is an anti-apoptosis function and promotes proliferation of cancer cells. OLFM4 regulates cell cycle and advanced S phase in cancer(13). Human OLFM4 is expressed in digestive system (stomach, colon and small intestine), prostate and bone marrow(15). The OLFM4 was found selectively in the epithelium of the crypt of the intestinal mucosa (16-18). The OLFM4 expression in the esophagus, stomach, small intestine and large intestine has also been observed by immunohistochemistry studies(19). Recently, evidence has been shown that OLFM4 expressed in several tumor types such as gastric tumors, pancreas, lungs, breast(20) and intestine.

COL11A1 gene encodes α1 chain of (pro)COL11A1 as well as mature COL11A1 which is an extracellular fibrillar collagen(21). Under normal situations, this gene and its derived products are mainly expressed by cartilage cells and mesenchymal stem cells and osteoblasts(22). Despite extensive expression of COL11A1 / (Pro) collagen 11A1 in active stromal cells in the desmoplastic process of many invasive human cancers(23), normal epithelial cells and quiescent fibroblasts do not express COL11A1 / (pro) COL11a in different regions(22). COL11A1 expression is highly correlated with the violence and progression of cancer and metastases in lymph nodes(24). It seems TGF-β1, Wnt and Hh signaling pathways regulate COL11A1 expression level. They are particularly active in stromal cell cancer (25-28). In our study we focused on the expression changing level of OLFM4 and COL11A1 in Iranian patients to identify role of these genes in gastric cancer progression. 

## Methods

**Sample collection**

 In this study, 25 GC tissue samples were compared with 10 normal stomach tissues. Samples were collected within two years (2014-2016) with collaboration of hospitals in Tehran and obtaining written consent from patients. Pathological and clinical examinations (type, site, size, depth of invasion, lymphatic invasion and progression) and data requirements were attained. Patients were between 27 to 84 years. Among them, there were 7 females and 18 males. 

Samples of tumor tissues were acquired surgically and pathological assessments were done for each one. Afterward, pieces of tumor tissues were selected and stored in RNA later as stabilization reagent (Qiagen, Germany) and were held in -70 fridge. Normal tissue samples were also collected endoscopically and kept in the same condition of tumor samples (18). 

**Table 1:** Primer sequences

**Gene**	**Primer**		**Amplicon**
OLFM4	F	TGCAGGGGTGTCTAAAAGTG	119
	R	AGGCAGATGATTCCCAAGAG	
COL11A1	F	TGGTTCAGTTGGTGGTGTTG	119
	R	CAGCTTCCCCTTTCTCTCCT	
GAPDH	F	AATCCCATCACCATCTTCCA	81
	R	TGGACTCCACGACGTACTCA	

**Figure 1:**
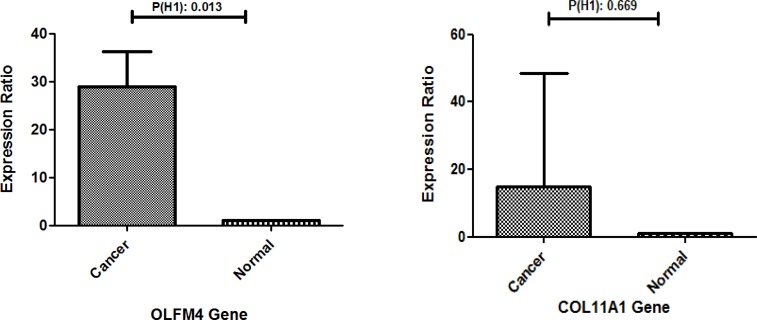
Expression ratio of OLFM4 and COL11A1. A) OLFM4 is UP-regulated in sample group (in comparison to control group ) by mean factor 5.318 (S.E range is 0.234 – 140.632). OLFM4 sample group is different to control group. P(H1) = 0.013 B) COL11A1 sample group is not different to control group. P(H1) = 0. 669

**Figure 2:**
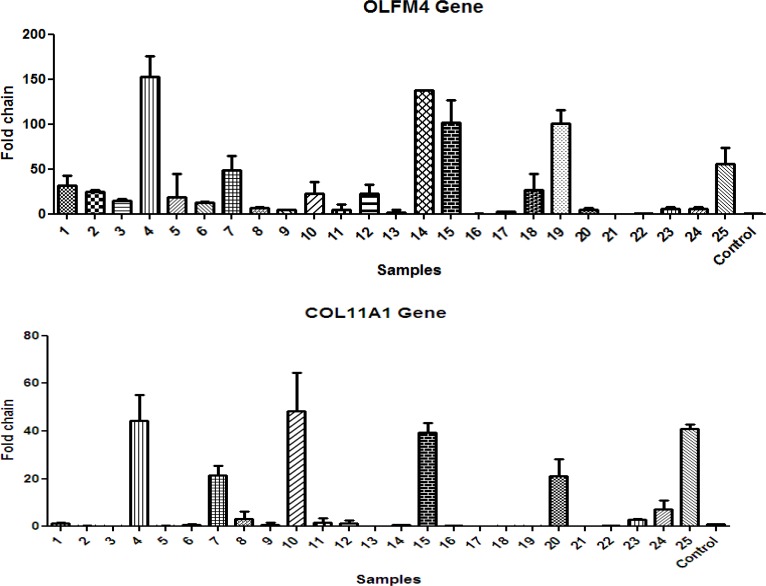
Expression level of OLFM4 and COL11A1 in patients in comparison to healthy individuals

**Total RNA extraction**

 Up to 50 mg of each sample was used for total RNA extraction. Based on the manufacturer’s instructions, RNA extraction were performed by RNeasy Plus Universal Mini kit (Qiagen, Germany). Quality and concentration of RNA extract were determined by NanoDrop 1000 (Thermo Fisher Scientific, USA). 

**cDNA synthesis**

 cDNA synthesis from RNA extraction with sufficient quality (260/280 more than 108) was served based on cDnA synthesis kit (Yekta tajhiz azma, Iran), M-MLV RT enzyme (Moloney murine leukemia Virus Reverse transcriptase) and random hexamer primer. Quality and concentration of synthesized cDNA was calculated by NanoDrop 1000. 

**Primer designing**

 Initially, mRNA sequence of OLFM4 and COL11A1 genes (target gene) and GAPDH (Glyceraldehyde-3-phosphate dehydrogenase) as reference gene were selected from NCBI. 

Primer designing was performed by www.primer3.com. Accuracy and sequence specificity of designed primers were checked in Gene Runner software using BLAST. Primer sequences are in Table 1. 

**Real time –PCR**

 OLFM4, COL11A1 and GAPDH genes were considered respectively as target and reference genes (gene of interest and housekeeping gene). Real time PCR cycler (rotor – gene Q model-QIAGEN) was used for RT – PCR. Based on MIQE protocol, SYBER-Green I, reverse and forward primers, cDNA and nuclease free water were applied for final valium of 20μl mixture reaction. Duplicate reactions along with no template control reactions were contemplated for 0.2 μl of each gene in every PCR tubes (QIAGEN, Germany). Temperature-time cycle was as follow, step1: temperature 95˚c for 30 second, step2: temperature 95˚c for 5 second and step3: temperature 60˚c for 34 second.

## Results

Data analysis were done by REST and GraphPad Prism software. It is demonstrated that in patients, OLFM4 expression levels are significantly (P<0.05) increased with the average of more than 5.318 fold in comparison to normal people. There were no significant differences in COL11A1 expression level in patients in comparison to healthy individuals (Figure 1 and 2). 

The results of Iranian patients with gastric cancer confirmed the findings of other countries about the overexpression of OLFM4 in gastric cancer tumors, which indicates that this gene can serve as a potential biological marker for prediction, diagnosis, and treatment of GC.

## Discussion

More than 952000 new GC cases have been reported in 2012 thus it is estimated as fifth most common cancer worldwide and also third lethal cancer in both genders(3). Since GC is a main fatal factor globally, it is more prevalent in East Asian countries such as China, Japan and Korea(29). 

Growth and development of GC are taking slowly over the years(30). Before the appearance of true-cancer symptoms, pre-cancerous changes occur in the lining of the stomach. As these early changes rarely cause symptoms, therefore it is often hard to identify(30).

Gastric cancer statistics are varying geographically. Thus national research could be more beneficial to express the condition of the disease inside the country(31). Over the past decades, there are some examinations focused on GC survival rate in Iran which are similar and relevant to developing countries and less than developed countries(32). According to recent reports, the survival rate of 1 to 5 years in patients with GC in Iran is 52%, 31%, 24%, 22%, 15%, respectively(1).

Given high mortality rate of disease, due to the fact that the apparent diagnosis of many gastric cancer patients is only possible when the cancer is in advanced stages, exactly when existing therapies are very limited. Therefore, identification of the involved mechanisms as well as biological markers are essential for developing an appropriate screening strategy, early detection, and treatment of gastric cancer(33).

In 1991, the first member of the olfactomedin family was discovered in bullfrog olfactory neuroepithelium and based on its localization, named Bolfactomedin(9).

The human OLFM4 gene, is located on chromosome 13q14.3 and encoding a 510 amino acid glycoprotein, which is then cloned from human hematopoietic myeloid cells(8).

Human olfactomedin 4 (OLFM4) has 65 % amino acid similarity to the canonical olfactomedin protein but has distinct tissue expression patterns and biological functions from other olfactomedin proteins (8, 12, 14, 34-36). This family are glycoproteins with a signal peptide, an N-terminal coilcoil, and a C-terminal olfactomedin domain. Phylogenetic analysis has recognized more than 100 olfactomedin domain containing conserved proteins in many multicellular organism species(37).

Evidence suggests that olfactomedin proteins are disease related and key regulators in a variety of biological functions(38). The encoded protein is an antiapoptotic factor that promotes tumor growth and is an extracellular matrix glycoprotein that facilitates cell adhesion(13). 

G-CSF was the first factor discovered to induce OLFM4 expression(8). Further studies have shown that different factors and signal pathways, including those that are important for inflammation and carcinogenesis, regulate OLFM4 transcription. These regulatory factors and pathways include PU.1, Retinoic acids, Estrogen and epidermal growth factor, MiR-486, NF-κB signaling pathway, Wnt signaling pathway, Notch signaling pathway(39). Genetic alterations of OLFM4 have been found in several different human cancers(40) such as colon cancer(41) and prostate cancer(42).

These findings suggest that OLFM4 could be a beneficial biomarker for some cancer precursors or early-stage cancers(43). Furthermore, genetic manipulation of OLFM4 expression levels could be a useful approach to prevent progression of some cancers, therefore benefiting some patients with those cancers(44).

Recent reports suggest OLFM4 as a novel marker for the differentiation(36), progression(45) and lymph node metastasis in gastric cancer(46).

Bo Gun Jung et al. has been reported previously, *Helicobacter pylori *induces OLFM4 expression through the NF-κB pathway, with a considerable effect on host defense against *H. pylori* infection (47-49). 

S Shinozaki et al. has stated that OLFM4 was the dominant type and their expression was restricted to the crypt epithelium(50).

*OLFM4* is a novel gene that has little homology to other known genes. It is overexpressed in a number of human tumor types, especially in those of the digestive system(51).

In current study, the OLFM4 expression level in Iranian patients with gastric cancer was evaluated by using the Real Time-PCR method. As expected, the results indicated upregulation of OLFM4 expression in tumor tissue compared to normal tissue in this country. Due to the fact that OLFM4 up-regulation expression level plays significant role in the cancer progression, measuring the expression level of this gene using a quantitative and accurate method, can have an effective role in reducing mortality in people with gastric cancer, and as a biomarker candidate, it could be considered in the diagnosis and cancer prevention treatments.

The human COL11A1 gene encodes the procollagen α1 chain and the mature collagen type XI, which is a small, extracellular fibrous collagen. Each collagen promoter is made up of three different polypeptides so-called α1, α2, α3 and is coded by specific gene sequences. In the XI collagen, the α1 and α2 chains are coded by the COL11A1 and COL11A2 sequences, respectively. While the α3 chain is quite similar to the α1 (II) chain, this chain is coded by the COL2A1 gene, which is the main component of the collagen type II(21, 52).

In order to a fibril formation, by the covalent interconnection between the telopeptides and the triple specific polypeptide helix transplantation regions, mature collagen molecules aggregate at the cell surface or in the extracellular matrix(21, 52).

Extracellular collagen, along with proteoglycans and glycoproteins such as fibronectin and tenascin C, etc., are the main ingredient of the extracellular matrix. In adults, (pro)COL11A1 is found in the eye, inner ear, clear cartilage, in the *nucleus pulposus *of the disc between the vertebrae(22). In the latter, most of the cartilage cells are made by the corneal fibroblasts (keratocytes) in the eye. It is also reported that it is made by mesenchymal and osteoblast stem cells. It has been reported that vascular smooth muscle cells are expressed COL11A1 as well(6). 

Under common regular conditions, COL11A1 / (pro) Col11A1 is not expressed in head, neck, breast, lung, stomach, uterus, pancreas, and large intestine stromal cells and it is present in almost all the benign pathologic processes such as chest hyperplasia, pulmonary adenosis(53), IPF, cirrhosis(54), diverticulitis, and inflammatory diseases(55).

COL11A1 / (pro) COL11A1 is overexpressed in activated stromal cells with desmoplastic reactions of severe human cancers(23). These cancers include oral / throat, head, and neck, breast, and lungs, esophagus, stomach, pancreas, colon, and ovary. In this case, COL11A1 / (pro) COL11A1 expression is associated with the cancer progression rate and metastases in the lymph nodes(22).

According to research conducted by Vecci et al., COL11A1 is a gene with advanced expression level in gastric cancer. On the other hand, COL11A1 gene expression level in the early stages of gastric cancer is not much increased. It is expected that it is possible to detect malignant and cancerous injuries with COL11A1(56).

Contrasting the results of present study for gene expression level of COL11A1 in Iran with the results of the previous researches in the western countries no significant difference for COL11A1 expression level in Iran was observed while it was increased in western countries which could be because of the geographical and environmental characteristics and also other effective reasons which should be investigated in the future researches.

The expression level of OLFM4 and COL11A1 are summarized in Figure 1. General comparison expression level of these two genes shows overexpression of OLFM4 in spite of COL11A1 in Iranian patients. Thus, OLFM4 can be used as a novel biomarker in gastric cancer recognition and progression in early stages. Moreover, OLFM4 can be useful in choosing appropriate treatment procedures based on the stage of the disease.

## Conflict of interests

The authors declare that they have no conflict of interest.
